# Genetics of Uveal Melanoma and Cutaneous Melanoma: Two of a Kind?

**DOI:** 10.1155/2010/360136

**Published:** 2010-06-06

**Authors:** Thomas van den Bosch, Emine Kilic, Dion Paridaens, Annelies de Klein

**Affiliations:** ^1^Department of Ocular Oncology, The Rotterdam Eye Hospital, The Netherlands; ^2^Department of Clinical Genetics, Erasmus Medical Center, P.O. Box 2040, 3000 CA Rotterdam, The Netherlands; ^3^Department of Ophthalmology, Erasmus Medical Center, P.O. Box 70030, 3000 LM Rotterdam, The Netherlands

## Abstract

Cutaneous melanoma and uveal melanoma both derive from melanocytes but show remarkable differences in tumorigenesis, mode of metastatic spread, genetic alterations, and therapeutic response. In this review we discuss the differences and similarities along with the genetic research techniques available and the contribution to our current understanding of melanoma. The several chromosomal aberrations already identified prove to be very strong predictors of decreased survival in CM and UM patients. Especially in UM, where the overall risk of metastasis is high (45%), genetic research might aid clinicians in selecting high-risk patients for future systemic adjuvant therapies.

## 1. Introduction

**Figure 1:**
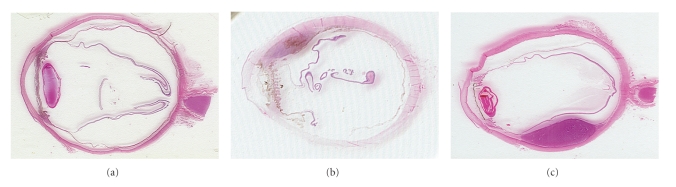
Uveal melanoma located in: iris (a), ciliary body (b) and choroid (c).

Cutaneous melanoma (CM) has shown to be one of the life-threatening malignancies with the fastest rise in incidence over the last decades. The highest incidence of CM is observed in Australia (60–70 per 100.000 individuals). In Europe and the USA the incidence is lower (10–15 and 20–30 per 100.000 [1, 2], resp.). CM accounts for more than 90% of all melanomas [3], whereas uveal melanoma (UM) is only encountered in 5% [4]. Nevertheless, UM is the most frequently occurring intraocular malignancy (85%) in the western world. Although CM and UM both derive from melanocytes, these two distinct tumors show remarkable differences in tumorigenesis, mode of metastatic spread, genetic alterations, and therapeutic response [5, 6]. CM can occur anywhere on the body but is predominantly observed in sun-exposed body parts. This partly explains the high incidence of CM in the light-skinned residents of Australia and New-Zealand. UM can occur anywhere along the uveal tract but tend to occur more frequently in the choroid (80%) and the ciliary body (15%) (Figure 1). The incidence of UM appears to be relatively stable with around 7 new patients per 1 million individuals yearly in the western world. UV-light exposure has shown not to be of specific risk in UM. However recently, Schmidt et al. [7] demonstrated a positive interaction between UM and individuals with light colored eyes who sustained frequent UV-radiation. In addition, the tendency of iris melanomas to occur in the lower half of the iris has been explained by the increased sunlight exposure of this area [8]. Other known risk factors for CM and UM are fair skin type (CM and UM), familial occurrence of melanoma (CM) [9], number of melanocytic naevi (CM), light colored eyes (UM), and oculodermal melanocytosis (UM) [10, 11].

## 2. Diagnostics

Clinical examination of suspicious lesions remains an important modality in diagnosing CM and UM. As for the diagnosis in CM, dermatologists rely mostly on clinical examination and reserve (excisional) biopsy for tumors of uncertain origin. Only UM of the iris can be diagnosed by external examination and is therefore detected in an early stage. For detection of UM of the choroid or ciliary body, a thorough ophthalmic examination including indirect ophthalmoscopy and ultrasonography of the retina has to be conducted. Tumor growth can lead to retinal detachment and result in extraocular extension of the tumor. At this point, defects in visual field or central vision may be present. Early symptoms of tumor growth, however, can be vague or absent to the patients' notion. 

The overall survival is known to be dependent of the tumor thickness (CM) and largest tumor diameter (UM) at time of diagnosis. Therefore, clinicians still concentrate on early detection of CM and UM. This resulted in an average tumor thickness of 0.76 mm in CM at time of diagnosis nowadays. This was shown to relate to an overall 10-year survival of 90% [12] of these small lesions. Similarly, UMs with a diameter of under 4 mm relate to a 5-year survival of 84%. The 5-year survival rate for medium-sized UM (4–8 mm in diameter) is 68%, and 47% with large size UM (over 8 mm in diameter) [13]. The survival of CM and UM patients with metastatic disease is however equally bad with a dismal mean of 2–7 months [14–16].

## 3. Therapy

The most frequently used therapeutic option in CM is excision of the primary tumor and enucleation of the tumor containing eye in case of large UM. Most small and medium-sized UMs are currently managed by eye-saving treatments such as observation (small inactive tumors), episcleral brachytherapy or charged-particle radiotherapy, and several other variants of radiotherapy. In CM, radiotherapy is only used for palliative purposes as CM cells appear to be relatively radio-resistant. Adjuvant systemic therapy is mainly used in patients at high-risk of metastasis or in patients who already have developed metastasis. The response rates of chemotherapeutic agents in metastasized CM and UM are however as low as 7%–25% [17–20].

## 4. Metastasis

Both malignancies display a strong tendency to metastasize [3]. Although the mode of metastatic spread is different, CMs tend to metastasize by both hematogenous and lymphogenous route and local invasion. CMs are known to be able to give rise to metastases in skin (13%–38%), distant lymph nodes (5%–34%), distant subcutaneous tissues (32%), lung (18%–36%), liver (14%–20%), CNS (2%–20%), and/or bone (4%–17%) [21]. In UM, metastatic spread is almost exclusively by hematogenous route to remote organs of which the liver is involved in almost all cases (90%) [15, 22]. The reason why UM is not involved in metastatic spread by lymphogenous route is thought to be a direct result of the absence of draining lymphatics of the eye [6, 23]. It is however still unknown why the liver is especially affected by metastases although there are reports about sporadic metastases in lung (24%) and bone (16%) [24–27].

Eventually 45% of UM patients die of metastasis regardless of enucleation or radiotherapy [16]. This has led to theories about the early presence of micrometastasis in the disease process, which remain dormant for years before they give rise to clinically detectable macrometastasis [28]. The exact duration of this proposed state of dormancy and cues for metastatic development remains uncertain. Shields et al. [29, 30] reported tumors with a size of just 1.0 mm to be capable of metastasizing, hence the need for highly specific and sensitive prognostic markers to predict which patient is at risk of developing metastasis. In the quest for significant prognostic markers in UM, already several have been identified. Age (over 60 years), largest basal tumor diameter (over 18 mm), tumor cell type (epitheloid cellularity), and closed vascular patterns correlate with early metastatic disease and shorter survival [31–33]. In CM, tumor thickness (increasing Breslow thicknesses), level of invasion, age (old age), gender (males), anatomic site of primary tumor (head/neck or trunk), number of metastatic lymph nodes, and ulceration on histopathological research appeared to be independent significant prognostic factors of early metastasis [21]. These factors are summarized in a staging system known as the TNM-staging system. This system relies on tumor stage at time of diagnosis which has shown to be the most important prognostic factor in CM and is now widely used for prognostic purposes and clinical decision making [34].

## 5. Tumor Research Methods

Genetic analysis of tumor material, either from excised CM or from enucleated eyes, has led to the identification of genetic prognostic markers for both types of melanoma.

In the past years several cytogenetic and molecular genetic techniques have been used to investigate the genomic background of melanomas. With conventional karyotyping, we and others were able to identify chromosomal gains, losses, and translocations in UM (Table 1). Comparative Genomic Hybridization (CGH) allows a complete copy number analysis of the entire genome by comparative hybridization of differentially labelled genomic sample and reference DNA to normal human metaphase spreads. Both these techniques have a low resolution of 5–20 MB. Fluorescence in situ Hybridisation (FISH) provides a higher test resolution and even clonal gains and losses present in only a low percentage of tumor cells can be detected [35, 36]. Furthermore, FISH has high test specificity, and although time consuming, still it is a frequently used technique in tumor research and diagnostics. Also paraffin-embedded tissue sections can be assayed by FISH. A drawback to this technique, however, is that only a small number of loci can be analyzed in one single experiment. Molecular genetic techniques such as multiplex ligation-dependent probe amplification (MLPA) and microsatellite instability analysis (MSI) require input of isolated DNA and enable analysis of multiple loci in one experiment with a high resolution. 

MLPA is a polymerase chain reaction- (PCR-) based technique which functions through the simultaneous hybridization of multiple (up to 50) probes to tumor DNA. Each probe with unique length is only amplified when ligated to its unique probe-counterpart. This provides high specificity of hybridized probes. The final amount of DNA, after several PCR-cycles, is dependent of its initial quantity and eventually copy number changes can be quantitated by relative quantification (RQ). MLPA has proven to be a suitable test for detection of chromosomal anomalies in tumor material [37].

For loss of heterozygosity (LOH) analysis, MSI is frequently used. With this technique, specific markers are required which are allowed to hybridize to the so-called microsatellites within genomic intronic DNA. These microsatellites are tandem repeats of simple polymorphic sequences that are randomly distributed and allow detection of the presence or absence of two different alleles. A drawback to this technique is that only a limited number of markers can be analyzed in a single experiment. 

Microarray-based CGH, single-nucleotide polymorphism (SNP) arrays, and gene expression analysis are among the most frequently applied array-based techniques nowadays. All these techniques are based on series of DNA segments (oligonucleotides or bacterial artificial chromosomes; BACs) orderly arranged on a chip, to which fluorescently labeled DNA or RNA can be hybridized. This enables the analysis of copy number status or gene expression of one entire genome very rapidly. Nowadays, there are chips available which enable analysis of structural variation at high level of detail with up to 1.2 million markers. The use of SNP arrays can also provide evaluation of loss of heterozygosity or isodisomy of parts of the genome.

Drawbacks to array technology are its cost, which is about tenfold compared to FISH, and the inability to detect balanced anomalies and genomic abnormalities in frequencies below 10% of analyzed nuclei. Table 1 provides an overview of the differences in resolution and detection limits among the cytogenetic and array-based techniques. The different research techniques previously mentioned certainly contributed to our understanding of melanoma by identification of chromosomes and genes involved in the disease. In the following section we will discuss the most important chromosomal and genetic alterations UM and CM.

## 6. Chromosomal Aberrations in UM

### 6.1. Chromosome 3

**Figure 2:**
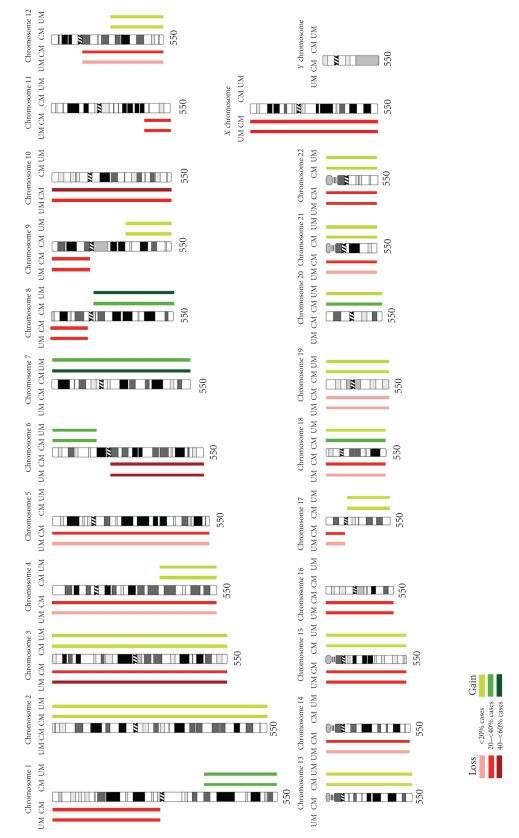
Chromosomal aberrations in cutaneous melanoma (CM) and uveal melanoma (UM): Based on all cases in the Mitelman Database of Chromosome Aberrations in cancer by Höglund [42].

The most frequently encountered chromosomal aberration in UM is loss of one of the two copies of chromosome 3 (monosomy 3 or −3). Monosomy 3 is observed in approximately 50% of cases [38–41] and appears rather specific for UM as this chromosomal anomaly is rarely encountered in CM or other cancer types [42] (Figure 2). Several groups have already shown that there is a strong correlation between monosomy 3 and the development of metastatic disease [43–46]. In addition, monosomy 3 strongly relates to several clinical and histopathological parameters such as epithelioid cytology, closed vascular patterns, large tumor diameter, and ciliary body involvement [41, 44, 47, 48]. Also, monosomy 3 is thought to represent an early event in tumorigenesis because the alteration is frequently seen in combination with all other known chromosomal abnormalities [49]. In 5%–10% of cases one copy of chromosome 3 is lost and the remaining copy is duplicated. This isodisomic state of chromosome 3 appears to be prognostically equivalent to monosomy 3 [50]. Rarely, partial deletions of chromosome 3 are found [26], and although this has hampered fine mapping studies, a common region of allelic loss on 3p25 and on 3q24–q26 could be defined [50, 51]. Most likely these regions harbor putative tumor suppressor genes but no specific genes have yet been identified. 

Gene expression profiling on UM tumor material does show promising results. By this technique UMs were found to cluster naturally in two distinct molecular classes (class I or class II) based on classifier gene sets [52]. Both classes appear to have clinical prognostic relevance; patients with class I tumors rarely die of metastases, while patients with class II tumors have a high risk of death due to metastases [33, 53]. Onken et al. [54] reported an eight-year survival of 95% for patients with class I UM and 31% for patients with class II UM. Moreover, class II tumors display the previously identified poor prognostic factors: monosomy 3, epitheloid cytology, and closed vascular patterns. The strong significant relation between molecular class and survival indicates that array technology clearly outperforms clinical and histopathological parameters [53–56].

### 6.2. Chromosome 8

Gain of 8q (+8q) is found in around 40% of UM cases and proved to be an independent significant prognostic marker for decreased survival [43, 45]. It frequently occurs in combination with monosomy 3, either as +8q or as isodisomy 8q, and this combination also shows a strong relation with metastatic disease [43–45, 57]. Abnormalities of chromosomes 3 and 8 are more common in ciliary body-located UMs; whilst alterations of the long arm of chromosome 8 tend to relate to choroid-derived UMs [43, 46, 48, 49]. However, in the study by Kilic et al. [58], chromosome 8q abnormalities were shown to correlate with large tumor diameter but there was no significant relation found between gain of 8q and the metastatic phenotype by univariate analysis. Gain of 8q is also frequently observed in different copy numbers in different UMs, therefore this is speculated to be a late event following the initiation of monosomy 3. The common region of amplification was found to range from 8q24.1 to 8q24.3 [59, 60]. Although gain of chromosome 8q is observed in 25% of CMs, the simultaneous occurrence of monosomy 3 and gain of 8q, as in UM, is rarely observed in CM. Several oncogenes on chromosome 8q were hinted as possible factors in UM pathogenesis; among these genes are *MYC* (on 8q24), *NBS*1 (on 8q21), and *DDEF*1(on 8q24) [46, 61–64]. A potential metastasis suppressor gene located on 8p21, named *LZTS*1, has been pointed out by Onken et al. [23].

### 6.3. Other Chromosomal Aberrations in UM

Kilic et al. [65] showed loss of 1p36 in combination with monosomy 3 to be of prognostic significance: these aberrations occurring together display a stronger correlation with decreased survival than monosomy 3 or loss of 1p36 alone (−1p36 by itself is not of prognostic significance). One of the suggested tumor suppressor genes in the 1p36 region, *APITD*1, was found to be not of significance in patients survival [66]. The common deleted regions on chromosome 1 were found to range from 1p34.3 to 36.2 [48, 67]. 

Alterations of chromosome 6 are frequently encountered in both UM & CM (discussed later) but show less prognostic value compared to monosomy 3 or gain of 8q in UM [42, 46]. Of these alterations, gain of DNA-material on the short arm of chromosome 6 (+6p) is found in 25%–29% of UM and relates to spindle cell cytology and low risk for development of metastasis [33, 42, 49, 68, 69]. Hughes et al. [60] reported the shortest region of overlap on the p-arm on chromosome 6 to be restricted to 6p22.3–p25. The simultaneous occurrence of +6p and −3, however, is rarely observed. Loss of DNA material on the long arm of chromosome 6 (−6q), observed in 25%–38%, possibly represents another late event in tumorigenesis and correlates with worse prognosis [39, 42, 48, 69, 70]. The region of common deletion on the long arm was found to range from 6q16.1 to 22.3 [60]. 

Infrequently, abnormalities of the other chromosomes such as loss of 9p, loss of chromosome 10, loss of 11q23–q25, and gain of chromosomes 7 and 10 have been reported [39, 40, 44, 46, 47] but a possible role in tumorigenesis and/ or development of metastasis in UM has yet to be evaluated.

### 6.4. Genes

Much less is known about genes involved in the development and progression to metastasis in UM compared to CM. This is mainly the result of the lower incidence of UM and the small quantities of tumor sample available for research. While there are many different potential tumor genes identified in CM every year, UM lags behind. However, several candidate genes were proposed in UM recently, such as *GNAQ, DDEF1, NBS1, HDM2, BCL-*2, and *CCND*1. For most of these genes, a definite role in tumorigenesis or progression towards metastasis has to be validated.

G protein alpha subunit q (*GNAQ*) is the first gene found to be mutated frequently in UM. Several groups have shown that approximately 46% of UMs carry mutations in the *GNAQ* gene [27, 81, 104] (Table 2) turning *GNAQ* into an oncogene. This oncogenic conversion leads to constitutive activation of the MAP-kinase pathway which results in a situation in which the cell is provided continuous growth signals in the absence of extracellular stimuli [113] and thus cell proliferation. *GNAQ* status was found not to be correlated with disease free survival; so it could represent an early event in tumorigenesis [27, 104]. This mutation is also found in 83% of blue naevi of the skin [81]. 

Furthermore, the *DDEF*1-gene has been described in UM. It is located on 8q24 and found to be mutated in 50% of UMs leading to overexpression [69]. High expression of *DDEF*1 was shown to result in more motile low-grade UM cells by Ehlers et al. [63] and could therefore be important in metastatic development [63, 114, 115]. The *NBS*1-gene is found to be overexpressed in 50% of UM [62]. The encoded protein product is postulated to be part of a complex involved in DNA-repair [102]. It is theorized that overexpressed *NBS*1 could allow UM progression by promoting the repair of DNA damage which occurs more frequently in advanced tumors with increased genetic instability. High expression of the *HDM*2-gene on 12q15 is found in 97% of UM [69]. High *HDM*2 expression was shown to inhibit p53 and its function of eliminating abnormal cells [105, 106]. An elevated expression of *BCL*-2, located on 18q21, is observed in UM but also in normal melanocytes. This overexpression is reported to block apoptosis [105, 106, 108, 109] and is suggested to be responsible for the resistance to chemotherapy or irradiation of melanocytes [69, 116]. In 65% of UM cases, *CCND*1 is reported to be overexpressed. Overexpression of *CCND*1 leads to activation of cyclin dependent kinases (CDKs) which consequently phosphorylate and inactivate Rb [69, 106, 107]. The *CCND*1 overexpression is associated with large tumor size, epitheloid cytology, and poor prognosis [106].

## 7. Chromosomal Aberrations in CM

CMs display a more complex karyotype compared to UM. The most frequently observed chromosomal aberration in CM is monosomy 10. This aberration is found in approximately 60% of CM cases and appears to be significantly more frequent compared to UM, where monosomy 10 is found in 27% of cases [42] (Figure 2). Because monosomy 10 could include loss of tumor suppressor genes, much research has been aimed at identifying possible tumor suppressor genes involved. Phosphatase and tensin homolog (*PTEN*) is one of the identified tumor suppressor genes, located on 10q23, with strong evidence for a role in CM tumorigenesis [92] (Table 2). *PTEN* is thought to be inactivated by deletion or mutation and through loss of its negative regulatory effect on AKT, lead to activation of the AKT-pathway, and consequently prevent apoptosis [85, 111]. The actual inactivation of *PTEN* is observed in up to 30%–40% of CM cell lines [92, 93], but only in 10% of primary CMs. *PTEN* inactivation or downregulation is mainly found in tumors with an increase in aneuploidy, suggesting that it is a late event in tumor progression [27, 111]. In UM, inactivation of *PTEN* is reported in 15% of cases and has been linked to an increase in aneuploidy but also poor clinical outcome [111, 112]. 

The other frequently reported chromosomal aberrations involved in CM are −1p, +1q, −4, −5, −6q, +7, −9p, −11q, −12q, −14, −15, −16, −17p, +18, +20, −21, and −22 [42]. Some of them will be discussed here along with the most well-known genes, involved in tumorigenesis and/ or metastatic development. 

### 7.1. Chromosome 1

Rearrangements of the distal part of the short arm of chromosome 1, leading to loss or gain of 1p, are reported in 28% and, respectively, 33% of CMs. Several regions along chromosome 1 are of specific interest because they harbor the *NRAS*- and *AKT*3-gene. *NRAS* is located in the 1p13-region and shown to be activated by mutation in 15%–25% of CMs [71, 72]. *NRAS* is believed to be also involved in the MAP-kinase pathway. Activation of *NRAS* leads to activation of the MAP-kinase pathway and as a result cellular proliferation. Additionally, *NRAS* binds and activates lipid kinase phosphoinositide-3 kinase (PI3K), thereby activating the AKT-pathway and preventing apoptosis [85]. A direct activating mutation of the *AKT*3-gene located on 1q44 is found in 40–67% of CMs [73]. Overexpression of *AKT*3 renders cells less sensitive to apoptotic stimuli and as mentioned before; *PTEN* inactivation can lead to the selective activation of AKT in CMs [92]. Different groups have shown *NRAS* mutations to be very rare in UM [95–98].

### 7.2. Chromosome 6

Alterations of chromosome 6 are reported in a total of 66% of CMs, of which +6p is observed in 24% and −6q in 42% [42]. Of these alterations, the 6q10–q27 region shows the highest frequency of rearrangements as a result of deletion, translocation, or due to the formation of an isochromosome of its short arm. The region on the short arm of chromosome 6 that frequently shows alterations spans from 6p21 to 6p25 and mainly results in gain of DNA material. Up till now, there have not been reports about possible over- or underexpressed genes on chromosome 6 involved in tumorigenesis. As mentioned before, both +6p and −6q are common in UM. The prognostic value of these alterations, however, proved to be lower than in CM [42, 46].

### 7.3. Chromosome 7

In 36% of CMs, gain of DNA-material on both arms of chromosome 7 is observed. Most frequently described are somatic mutations within the 7q34 region, where the *BRAF*-gene is located. Up to 60%–70% of CMs are characterized by activating mutations in *BRAF* [74]. The *BRAF*-gene encodes a kinase involved in the MAP-kinase pathway which, by mutation, is thought to lead to constitutive activation of the aforementioned pathway [117] and cell proliferation. A single substitution (p.V600E) appears to account for more than 90% of all *BRAF* mutations [118]. The same mutation is also found in 80% of benign naevi and is therefore believed to be an early event in melanomagenesis [75]. There is however evidence from another study that indicates a role in later stages of tumor growth and development [76]. Mutations of *BRAF* were shown to be absent in UMs [95, 99]. But in a small study, *BRAF* mutations were shown to occur in 48% of UM of the iris [100].

### 7.4. Chromosome 9

Chromosomal aberrations on chromosome 9 presenting as either deletions of the short arm, −9p10–24 (37% of CMs), or long arm, +9q22–34 (15% of CMs), have been reported. One of the best characterized genes in CM is *CDKN2*A, located on 9p21. Inactivating mutations, or loss, results in inactivation of the two encoding tumor suppressor genes *p16* and *p14*. Both genes were already related to high susceptibility for CM and were found in a total of 30%–80% of familial CM [88–90]. These mutations are however rarely observed in sporadic CM [87] or UM [110].

## 8. Epigenetics

Over the last years, there have been growing interest for the role of epigenetics in CM and UM pathogenesis and metastasis. The most well-known epigenetic features are methylation and microRNAs (miRNAs). Both act through different mechanisms by which they are thought to alter normal gene transcription. Methylation is frequently reported to induce silencing of certain genes by direct methylation of DNA strands or hypermethylation of specific promoters. Because human cancers are theorized to cause global demethylation and promoter hypermethylation, it is thought that this could lead to activation of imprinted genes and the inactivation of genes [119]. In CM, several genes commonly hypermethylated have been identified such as *RASSF*1A*, APC, PYCARD, RARB, MGMT, DAPK, *3-*OST*-2*, HOXB*13*, SYK, TIMP*3A*, CDKN*2A*, FHIT, SOCS*1*, SOCS*2, and* PTEN*. In UM, the studies regarding gene/promoter methylation status are still limited but *CDKN2*A is found to be methylated in 33% of cases [120, 121]. Similarly, *RASSF*1 appears to be methylated in 13%–70% [122] and *hTERT* in up to 52% [123]. It is not certain whether these methylated sites contribute to metastasis. 

MiRNAs have recently come to the attention because of their inhibitory effect on translation of mRNAs into proteins. Although there are limited studies available on the role of miRNAs, several miRNAs have been marked as possibly involved in UM tumorigenesis and/or metastasis such as *let-7b*, *miR18a*, *miR-199a*, *miR495*, *miR549,* and more [124, 125]. Worley et al. [124] and Radhakrishnan et al. [125] reported differentially expressed sets of miRNAs that could accurately distinguish two different classes with a low- and high- risk potential for metastatic disease. These miRNAs were shown to bind to genes often found to be deleted in UM such as 8p22, but also 13q and 17p. In CM, many different miRNAs have been identified such as *miR-137*, *miR-182*, *miR-221*, *miR-222,* and different subtypes of the *let-7* family [126–129]. These are thought to act as important factors in CM tumorigenesis and metastasis; further research is however required to analyse their exact role in CM.

## 9. The Relation between CM and UM

Although there are many differences between CM and UM, they do share some features. First of all, both tumors derive from neural crest melanocytes which migrated to the epidermic tissue or the eye. This common origin is still observed on morphologic and gross histopathologic research of tumor material from CM and UM. The chromosomal regions frequently observed to be amplificated or deleted in both melanotic tumors do resemble each other although the exact frequencies in which they occur differ. For instance, monosomy 3 is observed in around 50% of UMs and in 25% of CMs. The same holds for gene expression status: many of the genes found to be frequently overexpressed or underexpressed in CM are also observed in UM. Furthermore, both tumors are highly metastatic which is illustrated by the early initiation of metastases. UM, however, is not known to spread by lymphogenous route as CM is. This is an important difference and possibly due to the anatomical restrictions of the eye and the lymphatic system. Another difference concerns the role of UV-radiation, which appears to be an important risk factor for the development of CM but is not known as a risk factor for development of choroid-localized UM. There is however evidence regarding an interaction between UV-radiation and development of UM in the easily to sunlight exposed iris [7]. 

Maybe the two types of melanotic tumors are more similar than previously thought because of its common origin and the differences are merely a result of the exact location of the melanoma and its direct environment. Each location has its own array of carcinogens to which the tissue is exposed to. For instance, the retina is less intensely exposed to UV-radiation than the skin. The epithelial environment the cutaneous melanocytes reside in leads to the cells having more epithelial qualities of which downregulation of the molecule E-cadherin during local invasion is an example. Uveal melanocytes do not require this “mesenchymal to epithelial transition” because they are not in an epithelial environment. This could for part explain the differences in the spectrum of mutations between the two types of melanocytic tumors.

## 10. Conclusion

Despite all developments in diagnostics and therapeutics of primary UMs in the last 20–30 years, there have been no significant decrease in metastasis-related deaths [6, 130]. The prognosis for patients with metastasized disease still is 2–7 months, regardless of systemic therapy. This is probably due to the early initiation of metastasis in both CM and UM, which underlines the need for early prognostication. This could, at least for part, be achieved by continuing the search for prognostic factors in CM and UM through genetic research on tumor material. Genetic research has showed us that CM and UM have aberrations in common but that these differ in frequency between the two tumors. Even so, both express many of the same genes but not all. In CM, alterations of chromosomes 1, 6, 7, 9, 10, 14, 16, and 21 are frequently observed and already several candidate genes and proteins involved in the tumorigenesis of CM have been identified. UMs were shown to frequently display chromosomal aberrations on chromosomes 1, 3, and 8. Of these, monosomy 3, gain of 8q, and the combination of loss of 1p36 and monosomy 3 appeared to be significant prognostic factors for decreased survival; There have not been identified genes yet that are prognostically active in UM, and at this point developments in UM lag behind compared to CM. New insights in UM, however, came about by gene expression profiling of UMs which were shown to cluster naturally in two classes with different prognosis [52–54]. Generally, array technology has proven to outperform clinical and histopathological parameters in determining a patients' prognosis. This led to the frequent usage of gene expression testing in the current clinical setting in an attempt to identify high-risk patients. We do have to remind that we do not yet know whether monosomy 3 and classifier genes are truly involved in tumor progression and metastatic potential or that those are merely markers of the underlying cause. Additionally, we have to evaluate whether these results may aid clinicians in assessing eligibility of patients for future (adjuvant) systemic therapies. Most of the genetic research is conducted on relatively large UMs because small UMs are treated conservatively and this has biased UM research. Recent groups already reported about the suitability of fine needle aspiration biopsy in harvesting of tumor material from patients treated with eye-saving modalities [131–134]. Also for this diagnostic option we have to evaluate whether this will be beneficial for patient care and can lead to predictions about prognosis for the individual patient. Some genetic markers have already proven its value in predicting prognosis next to clinical and histopathological markers and could lead to selection for patient-tailored therapies in the near future. Also, the challenge will be to prove or disprove the cost-effectiveness of array technology and find additional genetic markers predictive of worse prognosis in CM and UM patients. Concluding, much information has been gained by genetic research of melanonomas and further research could augment our knowledge. Because there are similarities between the two tumors, research on one of two tumors could provide clues for research on the other. Epigenetics, the whole new field in genetic research, does look like a promising ally in our quest to understanding of pathogenesis and metastasis in CM and UM and might provide us with valuable prognostic information in the near future.

## Figures and Tables

**Table 1:** Overview of techniques used in (molecular) cytogenetics.

Method	Resolution	Provides genome wide testing?	Detection balanced anomalies?	Detection unbalanced anomalies?
Karyotype	~5–10 Mb	+	+	+
G-banding				
FISH	~100 kb	−	+	+
SKY	~1-2 Mb	+	+	+
MSI	<1 kb	−	−	+
CGH	~5–20 Mb	+	−	+
MLPA	~1–40 kb	−	−	+
SNP/ CGH array	>100 kb	+	−	+

**Table 2:** Commonest known genetic changes in CM and UM.

CM					
	Gene	Mechanism	Location	Cases (%)	Reference

Proto oncogenes	NRAS	mutation	1p13	15–25	[71, 72]
	AKT3	amplification	1q44	40–67	[73]
	BRAF	mutation	7q34	36–61	[74–76]
	NBS1	amplification	8q21	*	[77]
	MYC	amplification	8q24	1–40	[78–80]
	DDEF1	amplification	8q24	—	
	GNAQ	mutation	9p21	83^∗^1^^	[81]
	CCND1	amplification	11q13	6–44	[82–84]
	HDM2	amplification	12q15	—	
	BCL-2	amplification	18q21	>90%	[85, 86]

Tumor suppressor genes	LZTS1	deletion	8p21	—	
	CDKN2A-sporadic	deletion, mutation	9p21	*	[87]
	CDKN2A-familial	deletion, mutation	9p21	30–80	[88–91]
	PTEN	deletion, mutation	10q23	10–40	[92–94]

UM					

	Gene	Mechanism	Location	Cases (%)	Reference

Proto oncogenes	NRAS	mutation	1p13	*	[95–98]
	AKT3	amplification	1q44	—	
	BRAF	mutation	7q34	48^∗^2^^	[81, 99–101]
	NBS1	amplification	8q21	50	[62, 102]
	MYC	amplification	8q24	43	[103]
	DDEF1	amplification	8q24	50	[63, 69]
	GNAQ	mutation	9p21	46	[81, 104]
	CCND1	amplification	11q13	65	[69, 105–107]
	HDM2	amplification	12q15	97	[69, 105, 106]
	BCL-2	amplification	18q21	100	[105, 108, 109]

Tumor suppressor genes	LZTS1	deletion	8p21	—	[23]
	CDKN2A-sporadic	deletion, mutation	9p21	*	[110]
	CDKN2A-familial	deletion, mutation	9p21	*	[110]
	PTEN	deletion, mutation	10q23	15	[111, 112]

— no data available.

*Rarely observed or sporadic reports in literature.

^∗^1^^Observed in 83% of blue naevi.

^∗^2^^Observed in 48% of iris melanomas.
